# Is it time to consider population screening for fracture risk in postmenopausal women? A position paper from the International Osteoporosis Foundation Epidemiology/Quality of Life Working Group

**DOI:** 10.1007/s11657-022-01117-6

**Published:** 2022-06-28

**Authors:** P. Chotiyarnwong, E. V. McCloskey, N. C. Harvey, M. Lorentzon, D. Prieto-Alhambra, B. Abrahamsen, J. D. Adachi, F. Borgström, O. Bruyere, J. J. Carey, P. Clark, C. Cooper, E. M. Curtis, E. Dennison, M. Diaz-Curiel, H. P. Dimai, D. Grigorie, M. Hiligsmann, P. Khashayar, E. M. Lewiecki, P. Lips, R. S. Lorenc, S. Ortolani, A. Papaioannou, S. Silverman, M. Sosa, P. Szulc, K. A. Ward, N. Yoshimura, J. A. Kanis

**Affiliations:** 1 Department of Oncology & Metabolism, Mellanby Centre for Musculoskeletal Research, MRC Versus Arthritis Centre for Integrated Research in Musculoskeletal Ageing, University of Sheffield, Sheffield, UK; 2 Department of Orthopaedic Surgery, Faculty of Medicine, Siriraj Hospital, Mahidol University, Bangkok, Thailand; 3 Centre for Metabolic Bone Diseases, Northern General Hospital, University of Sheffield, Herries Road, Sheffield, S5 7AU UK; 4 MRC Lifecourse Epidemiology Centre, University of Southampton, Southampton, UK; 5 University of Gothenburg, Gothenburg, Sweden; 6 Australian Catholic University, Melbourne, Australia; 7 Oxford NIHR Biomedical Research Centre, University of Oxford, Windmill Road, Oxford, OX3 7LD UK; 8 GREMPAL (Grup de Recerca en Malalties Prevalents de L’Aparell Locomotor) Research Group, CIBERFes and Idiap Jordi Gol Primary Care Research Institute, Universitat Autònoma de Barcelona and Instituto de Salud Carlos III, Gran Via de Les Corts Catalanes, 591 Atico, 08007 Barcelona, Spain; 9 Department of Clinical Research, Odense Patient Data Exploratory Network, University of Southern Denmark, Odense, Denmark; 10 Department of Medicine, Holbæk Hospital, Holbæk, Denmark; 11 Department of Medicine, Michael G DeGroote School of Medicine, St Joseph’s Healthcare-McMaster University, Hamilton, ON Canada; 12 Quantify Research, Stockholm, Sweden; 13 Department of Learning, Informatics, Management and Ethics (LIME), Karolinska Institutet, Stockholm, Sweden; 14 WHO Collaborating Center for Public Health Aspects of Musculo-Skeletal Health and Ageing, Division of Public Health, Epidemiology and Health Economics, University of Liège, Liège, Belgium; 15 School of Medicine, National University of Ireland Galway, Galway, Ireland; 16 Department of Rheumatology, Galway University Hospitals, Galway, Ireland; 17 Clinical Epidemiology Unit of Hospital Infantil de México Federico Gómez-Faculty of Medicine, Universidad Nacional Autónoma de México, UNAM, Mexico City, Mexico; 18 Hospital Universitario Fundación Jiménez Díaz, Madrid, Spain; 19 Department of Internal Medicine, Division of Endocrinology and Diabetology, Medical University of Graz, Graz, Austria; 20 Carol Davila University of Medicine, Bucharest, Romania; 21 Department of Endocrinology & Bone Metabolism, National Institute of Endocrinology, Bucharest, Romania; 22 Department of Health Services Research, CAPHRI Care and Public Health Research Institute, Maastricht University, Maastricht, the Netherlands; 23 Center for Microsystems Technology, Imec and Ghent University, 9050 Ghent, Belgium; 24 New Mexico Clinical Research & Osteoporosis Center, Albuquerque, NM USA; 25 Department of Internal Medicine, Endocrine Section & Amsterdam Public Health Research Institute, Amsterdam UMC, Vrije Universiteit Amsterdam, Amsterdam, the Netherlands; 26 Multidisciplinary Osteoporosis Forum, SOMED, Warsaw, Poland; 27 IRCCS Istituto Auxologico, UO Endocrinologia E Malattie del Metabolismo, Milano, Italy; 28 Department of Medicine, McMaster University, Hamilton, ON Canada; 29 GERAS Centre for Aging Research, Hamilton, ON Canada; 30 Cedars-Sinai Medical Center, Los Angeles, CA USA; 31 Bone Metabolic Unit, University of Las Palmas de Gran Canaria, Hospital University Insular, Las Palmas, Gran Canaria Spain; 32 INSERM UMR 1033, University of Lyon, Hôpital Edouard Herriot, Lyon, France; 33 Department of Preventive Medicine for Locomotive Organ Disorders, 22Nd Century Medical and Research Center, University of Tokyo, Hongo 7-3-1, Bunkyo-ku, Tokyo, 113-8655 Japan

**Keywords:** Fracture risk, Screening, FRAX, Treatment, Cost-effectiveness

## Abstract

***Summary*:**

The IOF Epidemiology and Quality of Life Working Group has reviewed the potential role of population screening for high hip fracture risk against well-established criteria. The report concludes that such an approach should strongly be considered in many health care systems to reduce the burden of hip fractures.

**Introduction:**

The burden of long-term osteoporosis management falls on primary care in most healthcare systems. However, a wide and stable treatment gap exists in many such settings; most of which appears to be secondary to a lack of awareness of fracture risk. Screening is a public health measure for the purpose of identifying individuals who are likely to benefit from further investigations and/or treatment to reduce the risk of a disease or its complications. The purpose of this report was to review the evidence for a potential screening programme to identify postmenopausal women at increased risk of hip fracture.

**Methods:**

The approach took well-established criteria for the development of a screening program, adapted by the UK National Screening Committee, and sought the opinion of 20 members of the International Osteoporosis Foundation’s Working Group on Epidemiology and Quality of Life as to whether each criterion was met (yes, partial or no). For each criterion, the evidence base was then reviewed and summarized.

**Results and Conclusion:**

The report concludes that evidence supports the proposal that screening for high fracture risk in primary care should strongly be considered for incorporation into many health care systems to reduce the burden of fractures, particularly hip fractures. The key remaining hurdles to overcome are engagement with primary care healthcare professionals, and the implementation of systems that facilitate and maintain the screening program.

## Introduction

In most countries, screening is regarded as a public health measure, the purpose of which is to offer a test to identify those individuals who are more likely to benefit from further investigations and/or treatment to reduce the risk of a disease or its complications. The test is targeted at a defined population, the members of which do not necessarily perceive that they are at risk of, or are already affected by, the disease or its complications. Principles to determine whether the approaches to managing a disease should include a screening programme were first proposed over 50 years ago [1] (Table 1).

**Table 1:** The original ten principles for a screening programme outlined by Wilson and Jungner [1]

1	The condition sought should be an important health problem
2	There should be an accepted treatment for patients with recognized disease
3	Facilities for diagnosis and treatment should be available
4	There should be a recognizable latent or early symptomatic stage
5	There should be a suitable test or examination
6	The test should be acceptable to the population
7	The natural history of the condition, including development from latent to declared disease, should be adequately understood
8	There should be an agreed policy on whom to treat as patients
9	The cost of case-finding (including diagnosis and treatment of patients diagnosed) should be economically balanced in relation to possible expenditure on medical care as a whole
10	Case-finding should be a continuing process and not a “once and for all” project

These principles have remained largely intact since then, with some modification by national and regional screening committees. For example, prior to making a formal assessment of a screening program, the UK National Screening Committee (NSC) examines certain general characteristics, unrelated to the specific disease, to guide decision making (Table 5). These include that the target population to be screened should be sufficiently large to enable safe, clinically and cost-effective screening, and that the population to be screened would regard themselves as not necessarily having symptoms of the disease or to be at risk of the disease (i.e. relatively healthy people). There should exist an effective means of identifying and contacting the whole cohort to be offered screening, including proactive approaches (e.g. written invitation, verbal invitation at other appointments), with those approached properly informed of the potential benefits and risks to make an informed choice. If these characteristics can be satisfied, the subsequent formal assessment of the evidence for screening covers the key issues, identified by Wilson and Jungner, relating to the condition, the test, the treatment and the effectiveness of any proposed screening programme. Several further aspects, considered by the UK NSC, are beyond the scope of this report: these include plans for managing and monitoring the screening programme, agreed quality assurance standards, adequate levels of staffing and facilities, provision of information to potential participants and anticipated public pressure to extend the eligibility criteria for screening.

As described below, osteoporosis and, more pertinently, its burden of fractures represents an opportunity for consideration of a screening strategy. The purpose of this paper is to propose a screening strategy for fracture risk reduction in postmenopausal women and to examine key issues in relation to this strategy.

## The proposed screening strategy

Given the effectiveness and cost-effectiveness of the intervention described later in this document, the strategy is based on the approach undertaken in the Screening for Osteoporosis in Older People (SCOOP) study in the UK [2–6]. In brief, a risk factor questionnaire based on the FRAX® risk assessment tool would be completed, in paper form or electronically, by women age 70 years or older through self-completion or completion assisted by a family member or caregiver. The questionnaire data would then be utilized centrally to calculate the 10-year major osteoporotic fracture probability and the 10-year hip fracture probability. Those with a low risk of hip fracture would receive a letter of reassurance with general lifestyle advice, while the remainder would have an additional assessment of femoral neck bone mineral density (BMD) using local densitometer facilities. The bone density result would then be incorporated in an updated FRAX calculation, with those that have hip fracture probabilities above the intervention threshold being recommended for treatment. The latter recommendation would be communicated to both the individual and their general practitioner.

As the effectiveness of any screening programme is not only dependent on the screening test but also on the prevalence of the disease of interest, we envisage that the strategy should initially be considered in older age groups, for example those age 70 years and above as in the SCOOP trial, in those countries with a hip fracture risk comparable to or higher than that in the UK [7].

## Methods — survey and responses

The opinion of 29 individual expert members of the Epidemiology and Quality of Life (EpiQOL) Working Group on whether screening for high hip fracture risk could fulfil each of the UK NSC criteria (Table 5) for a screening programme was canvassed using a Google Form. The survey was limited to the first 15 criteria as the final four address criteria that focus on issues around implementation of an accepted screening strategy. For each criterion, the experts were asked to say if the proposed strategy fulfilled that criterion (yes, in part or no) and could expand on their classification in a free text field.

Responses were received from 20 experts (69% of those approached), and the overall scores for each criterion are included in Table 5. Their feedback was incorporated into each of the sections below.

## Results-Criterion review

### The condition

#### The condition sought should be an important health problem

Osteoporosis is a systemic skeletal disease, characterised by low bone mass and microarchitectural deterioration of bone tissue with a subsequent increase in bone fragility and susceptibility to fracture [8]. The most serious clinical consequence of osteoporosis is hip fracture, though other fractures commonly occur at the spine, forearm and shoulder, and are often grouped together with hip fractures as major osteoporotic fractures.

Osteoporotic fractures are undoubtedly a common public health problem, particularly in ageing societies in terms of patient’s health, quality of life and social care costs [9–12]. At the age of 50 years, the remaining lifetime probability of at least one of the major osteoporotic fractures is 22% in men and 46% in women. In 2000, it was estimated that there were approximately 9 million fractures annually worldwide, with over one-third of all osteoporotic fractures occurring in Europe [13]. The latter accounted for 2 million disability-adjusted life years (DALYs) annually in Europe, a burden that exceeded that of hypertensive heart disease or rheumatoid arthritis. The number and burden of osteoporotic fractures is rising in many countries, partly related to the increased longevity of the populations. The age- and sex-specific incidence of fracture has also increased in some but not all countries [14, 15].

In 2019, 4.3 million new fragility fractures were estimated to have occurred in the EU, comprising approximately 827,000 hip fractures, 663,000 clinical vertebral (spine) fractures, 637,000 forearm fractures and 2,150,000 fractures at other sites (i.e. pelvis, rib, humerus, tibia, fibula, clavicle, scapula, sternum and other femoral fractures) [11]. About two-thirds of all incident fractures occurred in women. In 2019, the number of deaths causally related to fractures was estimated to be 248,487, with about half of these attributable to hip fractures. The number of fracture-related deaths was comparable or exceeded those from some of the most common causes of death such as lung cancer, diabetes and chronic lower respiratory diseases. In 2000, it was estimated that osteoporotic fractures in Europe accounted for more DALYs (2,006,000) than rheumatoid arthritis (1,048,000), but less than for osteoarthritis (3,088,000) [13]. In 2016, the estimate placed the total DALY loss related to fragility fractures at more than 2.6 million, which was higher than that estimated for chronic obstructive pulmonary disease and ischaemic stroke, but lower than that for lung cancer or dementia [12].

In 2019, the cost of osteoporosis, including pharmacological intervention in the EU was estimated at €56.9 billion (1000 million), with two-thirds derived from the treatment of fractures and only 3% representing the costs of pharmacological intervention. Excluding the latter cost, hip fractures represented 54% of the costs. The total cost including values of quality adjusted life years (QALYs) lost was estimated at €113 billion, a figure that is expected to rise to €121 billion in 2025. The cost of osteoporotic fractures accounts for approximately 3.5% of healthcare spending (i.e. €55.3 billion in 2019) indicating a very substantial impact of fragility fractures on the present healthcare budgets of European countries.

The epidemiology of osteoporosis is well characterized and described in detail elsewhere [14, 16–19]. The incidence of fragility fractures increases markedly with age, and the vast majority of osteoporotic fractures occur in older women. Compared with other fractures, a great deal of information is available on the epidemiology of hip fracture as nearly all such patients are admitted to hospital with data available through surgical and discharge records. In patients with fractures at other skeletal sites, only a minority are admitted, though they may attend hospital on an outpatient basis.

The occurrence of hip fracture, widely regarded as the most serious osteoporotic fracture, reflects decreasing bone strength with increasing age, with a concomitant increase in falls risk [20]. The risk of falling is somewhat higher in elderly women than in elderly men with about one-third of elderly individuals falling annually [21]. Hip fractures are relatively rare at the age of 50 years but become the predominant fracture from the age of 75 years [22]. Patients with hip fracture often have significant co-morbidities, and up to 20% of patients die in the first year following hip fracture, mostly as a result of these underlying medical conditions [23, 24]; nonetheless, it is estimated that approximately 30% of deaths are causally related to the fracture event [25].

In summary, there is ample evidence that osteoporosis and, in particular, osteoporosis-related fractures are a significant and growing health problem for individuals and healthcare providers. Fractures are highly prevalent and are associated with significant morbidity and mortality.

#### The epidemiology and natural history of the condition, including development from latent to declared disease, should be adequately understood and there should be a detectable risk factor, disease marker, latent period or early symptomatic stage

Clinically, bone health is most widely assessed by the non-invasive measurement of BMD by dual-energy x-ray absorptiometry (DXA) as there is a very well-established inverse correlation between BMD and future fracture risk [26]. As BMD is continuously distributed in the population, a WHO Scientific Group in 1994 developed a densitometric definition of osteoporosis using a threshold of 2.5 standard deviations below the mean BMD in young people of the same sex [27, 28]. Since then, osteoporosis has been operationally defined on the basis of BMD assessment, with a subsequent refinement to focus on measurements at the femoral neck [29]. This definition, however, has high specificity but low sensitivity for the prediction of fracture, as the majority of osteoporotic fractures occur in individuals with BMD values above the osteoporosis threshold [27]. This is the main reason that BMD measurement alone has not been accepted as a public health screening test in many countries to date, though there are notable exceptions including the USA [30, 31].

As a clinical endpoint, osteoporotic fracture has multiple factors contributing to the risk of fracture that can enable identification of high risk prior to the event occurring. In many ways, this is a different latency to that identified under established screening programmes in diseases such as breast and cervical cancer or diabetic eye disease, where the focus is on early detection of the disease itself. At a simplistic level, it might be assumed that low or decreasing bone mass is the only latent stage of note for osteoporotic fracture but, as described below, this excludes a wealth of information on other factors that contribute to fracture risk. This awareness has led to the development of risk calculators that take account of several risk indicators as used in the management of other diseases such as cardiovascular disease [32–34].

Examples of ‘non-BMD’ factors that contribute to fracture risk include ethnicity, age, sex, body mass index (BMI) [35], a prior fracture [36], a family history of fracture [37], long-term use of systemic glucocorticoids [38], a history of rheumatoid arthritis [39], diabetes [40–42], other causes of secondary osteoporosis [43] and lifestyle risk factors such as physical inactivity [44], falls [45, 46], smoking [47] and alcohol consumption[48]. It is important to note that some of these risk factors are partially or wholly independent of BMD, so that their use with BMD can enhance the information provided by BMD alone. Conversely, some strong BMD-dependent risk factors, for example BMI, can be used for fracture risk assessment in the absence of access to BMD tests. This is discussed in more detail in the section on the test.

In summary, the epidemiology and natural history of osteoporosis, including the risk factors that contribute to the clinical endpoint of fracture, are well documented and understood. The recognition of identifiable risk factors and the quantitation of that risk are now possible allowing preventative interventions to be targeted to those at highest fracture risk.

#### All the cost-effective primary prevention interventions should have been implemented as far as practicable

To date, there is no evidence base to demonstrate that cost-effective (and indeed effective) primary prevention interventions of a non-pharmacological nature reduce the burden of osteoporotic fractures. Where appropriate, lifestyle modifications are recommended comprising advice about nutrition (adequate calcium, vitamin D and protein intake), physical activity (weight-bearing, muscle strengthening and balance training exercise) and risk factor reduction (e.g. smoking cessation, alcohol intake reduction, vision correction, avoidance or reduction in exposure to drugs associated with osteoporosis). In general, a daily dietary calcium intake of around 800–1200 mg is recommended, with calcium supplementation where this cannot be achieved [49–54]. National recommendations for vitamin D supplementation vary according to the target population (whole population or subsets), the required dose and the target level of vitamin D as assessed by serum or plasma 25-hydroxyvitamin D [55–58]. The impact of combined calcium and vitamin D supplementation on fracture risk remains uncertain, particularly in the community-dwelling elderly [59–61].

Thus, whereas patients will continue to receive general advice in the absence of cost-effective primary prevention interventions, there remains a need to implement other proven, cost-effective strategies and interventions.

### The test

#### There should be a simple, safe, precise and validated screening test

The increasing recognition and acceptance that treatment for osteoporosis should be targeted on the basis of fracture risk requires well-validated assessment tools providing ease of use in clinical practice. Over the last 15 years, three tools have garnered interest, namely the FRAX® tool [62, 63], QFracture [64, 65] and the Garvan tool [66]. Of these, the FRAX tool has achieved widespread use with incorporation into numerous guidelines worldwide, with a large number of studies evaluating its utility [67]. In contrast to many risk calculators, both in the field of fracture risk or other disease areas, FRAX is unusual in that the output from the calculation is calibrated to the epidemiology of fracture and mortality for each country/region/ethnicity with an existing FRAX model. In short, this means that if the whole population were to undergo FRAX assessments, then the number of fractures predicted would be the number observed. This construct has been used to develop simulated population cohorts in the UK and elsewhere and has provided a basis for estimating the global burden of high fracture probabilities [18, 68, 69]. Most importantly, in the context of this review, FRAX is the only fracture risk assessment tool to be studied to date in randomised, controlled studies of population-based screening.

**Fig. 1:**
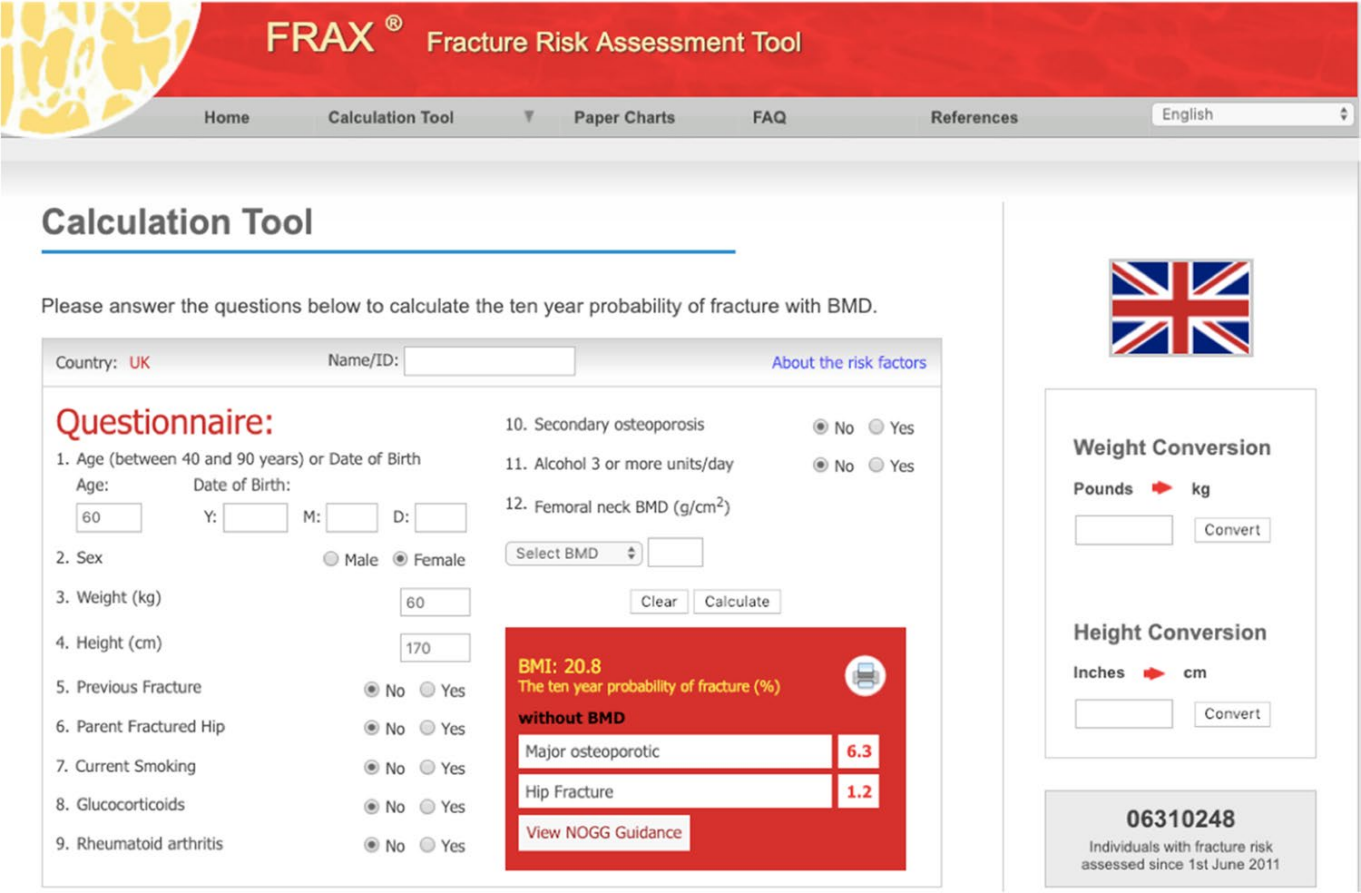
An example of the FRAX® fracture risk assessment tool webpage for the UK FRAX calculator. The UK calculator is linked through to the guidance pages of the National Osteoporosis Guideline Group (View NOGG Guidance button)

In brief, the FRAX® tool (https://www.sheffield.ac.uk/FRAX/index.aspx) is a computer-based algorithm developed by researchers at the then WHO Collaborating Centre for Metabolic Bone Diseases at the University of Sheffield [43, 70, 71]. The calculation incorporates information gathered from easily obtained clinical risk factors (Fig. 1) to estimate the probability of sustaining a fracture in the next 10 years. Femoral neck BMD can also be added to the calculation to enhance fracture risk prediction. It has two outputs, namely the probability of sustaining a hip fracture in the next 10 years, or a major osteoporotic fracture. The outputs are calibrated to age and sex-specific rates of fracture and mortality in a substantial number of countries/territories (currently 85 models covering 77 countries).

FRAX has been widely used for the assessment of fracture risk since the launch of the FRAX website in 2008. Following regulatory review by the US Food and Drug Administration, FRAX was incorporated into DXA scanners to provide FRAX probabilities at the time of DXA scanning. For those without internet access, hand-held calculators and an application for Apple smartphones have been developed. A paper-based FRAX pad in several languages allows patients to document risk variables prior to a visit with healthcare professionals.

It is also worthy of note that the availability and access to densitometry in many countries is low [72–75], so that a major advantage of FRAX is the ability to assess fracture risk where BMD is unavailable. In the UK, National Institute for Health and Care Excellence (NICE) has recommended that FRAX (or QFracture) should be used to estimate 10-year predicted absolute fracture risk prior to deciding on the need for DXA measured BMD [76]. As a completely non-invasive test, the tool is safe for users.

The characteristic of major importance, for the purpose of risk assessment, is the ability of a tool to predict the occurrence of new fractures, more usefully expressed as the increase in relative risk per standard deviation (SD) unit increase in risk score, termed the gradient of risk. The gradient of risk for the use of the FRAX clinical risk factors alone, femoral neck BMD alone, and the combination is shown in Table 2 [77]. It is relevant that the performance of FRAX is enhanced by the use of BMD tests; FRAX without BMD has a predictive value for fractures that is comparable to the use of BMD alone. Overall, the predictive value compares very favourably with other risk engines such as the Gail score for breast cancer [78]. The choice of screening modality to identify high hip fracture risk can be adapted to local resources; for example, where BMD measurements are readily available, it can play a predominant role in screening, while screening with FRAX risk factors alone can readily be applied in the absence of access to BMD.

**Table 2:** Gradients of risk (RR per SD change in with 95% confidence intervals) with the use of BMD at the femoral neck, FRAX clinical risk factors or the combination for hip and other osteoporotic fractures (not confined to major osteoporotic fractures). With kind permission from Springer Science + Business Media B.V] [77]

Age (years)	Gradient of risk		
	BMD only	Clinical risk factors alone	Clinical risk factors + BMD
*(a) Hip fracture*			
50	3.68 (2.61–5.19)	2.05 (1.58–2.65)	4.23 (3.12–5.73)
60	3.07 (2.42–3.89)	1.95 (1.63–2.33)	3.51 (2.85–4.33)
70	2.78 (2.39–3.23)	1.84 (1.65–2.05)	2.91 (2.56–3.31)
80	2.28 (2.09–2.50)	1.75 (1.62–1.90)	2.42 (2.18–2.69)
90	1.70 (1.50–1.93)	1.66 (1.47–1.87)	2.02 (1.71–2.38)
*(b) Other osteoporotic fractures*			
50	1.19 (1.05–1.34)	1.41 (1.28–1.56)	1.44 (1.30–1.59)
60	1.28 (1.18–1.39)	1.48 (1.39–1.58)	1.52 (1.42–1.62)
70	1.39 (1.30–1.48)	1.55 (1.48–1.62)	1.61 (1.54–1.68)
80	1.54 (1.44–1.65)	1.63 (1.54–1.72)	1.71 (1.62–1.80)
90	1.56 (1.40–1.75)	1.72 (1.58–1.88)	1.81 (1.67–1.97)

The performance characteristics of the FRAX clinical risk factors, with and without BMD, have been validated in eleven independent population-based cohorts [77]. Notably, the gradients of risk and age-adjusted area under the curves (AUC) from receiver-operated characteristic (ROC) analysis were comparable in the validation cohorts compared with the original cohorts. In contrast to separate analyses of sensitivity and specificity, the AUC provides an index of overall test accuracy if sensitivity and specificity have equal weights. A test with no predictive (or discriminative) ability would have an AUC of 0.5 (or 50%), while a perfect predictive test would have an AUC of 1.0 (or 100%). For hip fracture prediction without BMD, the mean AUC at age 70 years was 0.66 in the validation cohorts compared with 0.67 in the original cohorts. With the addition of BMD, the mean AUC was 0.74 and 0.78, respectively [77]. Since that original validation, a number of independent reviews, including four systematic reviews, have included detailed descriptions of the performance of the FRAX tool [76, 79–82]. Bearing in mind the flaws that can arise from cross-study and within-study comparisons of the AUC [83, 84], the reviews have confirmed a significant predictive ability of FRAX for future fractures, especially of the hip. For example, in the most recent of the systematic reviews, conducted for the US Preventive Services Task Force, the AUCs for hip fracture prediction with FRAX ranged from 0.76, in 12 studies comprising just under 200,000 women where FRAX was calculated without BMD, to 0.79 in 10 studies comprising approximately 162,000 women when FRAX was calculated with BMD [79]. In a recent independent comparative study in a single population sample, the predictive value for hip fractures was better for the FRAX tool (AUC: 0.841, 95% CI 0.795–0.887) than for the Garvan fracture risk tool (AUC: 0.769, 95% CI 0.702–0.836, *p* = 0.01) [85].

In summary, the FRAX tool fulfils the criteria of being a simple, safe, precise and well-validated test. While the AUCs for future hip fracture prediction are perhaps lower than existing screening tests that detect existing or early disease, they suggest excellent performance suitable for application in clinical practice.

The distribution of test values in the target population should be known and a suitable cut-off level defined and agreed.

Screening programmes are usually designed and adopted at regional or national levels. The same is true for the development and setting of intervention thresholds; this necessarily remains a more localised remit, given that each health care system will consider local/national factors such as reimbursement issues, health economic assessment, willingness to pay for health care in osteoporosis and access to DXA. The use of health economic analyses to derive intervention thresholds is fraught with problems, the most common of which is that they are time limited due to changes, usually reductions, in the costs of treatment. In the presence of very inexpensive medications, absurd situations can arise with a recent example from the UK where, following a multiple technology appraisal (MTA) on bisphosphonate use in osteoporosis, NICE recommended that treatment with oral bisphosphonates may be instituted in those with a 10-year probability of major osteoporotic fracture of 1% or more [86]. If interpreted as an intervention threshold, virtually all women aged ≥ 65 and men ≥ 75 years would be recommended treatment [87]. Shortly thereafter, NICE endorsed the assessment and intervention thresholds proposed within the NOGG guidance [50, 88].

A systematic review in 2016 identified assessment guidelines for osteoporosis that incorporated FRAX, utilising either age-independent (i.e. fixed) or age-dependent thresholds for further assessment and/or treatment [67]. In most guidelines, treatment for osteoporosis is recommended in individuals with prior fragility fractures, especially fractures at the spine or hip, with the FRAX thresholds reserved specifically for use in those without such fractures. The majority of guidelines had utilised a fixed intervention threshold, frequently as a component of more complex guidance (e.g. BMD thresholds). A substantial proportion (about 25%) had adopted an age-dependent threshold, with a small minority proposing a combination of age-dependent and fixed thresholds (so called hybrid thresholds).

Age-dependent intervention thresholds, first developed by the National Osteoporosis Guideline Group (NOGG), are based on the rationale that if a woman with a prior fragility fracture is eligible for treatment, then, at any given age, a man or woman with the same fracture probability (i.e. at the ‘fracture threshold’) should also be eligible, even in the absence of fracture [50, 89, 90]. By design, this fracture threshold increases with age with a plateau or decline at older ages due to the competing hazard of death. Age-dependent thresholds have since been adopted into European guidelines [51, 91] and elsewhere [67, 92, 93]. The same intervention threshold is applied to men, since the effectiveness and cost-effectiveness of interventions in men are broadly similar to that in women for equivalent risk [94]. In the UK, it was noted that the age-dependent probability thresholds introduced inequalities in access to therapy at older ages (≥ 70 years) for women without a prior fracture, leading to the development of a hybrid model which reduced the disparity [69]. For the proposed screening strategy, the approach would be to adopt the current NOGG thresholds in the UK. The thresholds at age 70 years for major osteoporotic fracture and hip fracture in the UK and some other high-risk countries, if using a similar approach, are shown in Table 3. Other countries that have an average 10-year hip fracture probability threshold of 5% or more include Austria, Denmark, Germany, Greece, Iceland, Iran, Ireland, Israel, Italy, Kazakhstan, Kyrgystan, Malta, Moldova, Norway, Singapore, Slovakia, South Korea, Sweden, Switzerland, Taiwan and Uzbekistan.

**Table 3:** Possible FRAX-based intervention thresholds in examples of high fracture risk countries if using the same approach as NOGG in the UK (ranked in descending order of hip fracture probability). Values represent FRAX 10-year probabilities of major osteoporotic (MOF) and hip fractures in women at the age of 70 years with a history of prior fracture and no other risk factors (BMI set to 25 kg/m^2^)

Country	Putative MOF probability threshold (%)	Putative hip probability threshold (%)
Denmark	28	8.8
Sweden	25	8.7
Norway	22	7.4
Singapore (Chinese)	19	6.0
USA (Caucasian)	21	5.0
UK	20	4.8
Canada	19	4.4
Japan	18	3.9

In summary, there are a number of approaches to defining cut-off levels for the FRAX tool as intervention thresholds within a screening program. Of these, the probability thresholds at age 70 in women with a prior fracture seem intuitive, as all current guidelines would recommend treatment in such individuals.

#### The test should be acceptable to the population

The field of research into acceptability of healthcare interventions is, perhaps surprisingly, underdeveloped [95]. DXA-based measurement of BMD is a noninvasive procedure, and the level of radiation exposure is very low and considered to be safe for the population [96]. Access to, and use of, the FRAX online tool suggests that it is viewed to have acceptable clinical utility by health professionals [67, 97, 98], though this has not been formally assessed. In the setting of primary prevention, a formal assessment has been undertaken as part of the SCOOP study [2]. This qualitative sub-study sought to capture the views of older women and GPs about the acceptability of screening, using the FRAX questionnaire with BMD in those at medium to high risk, to prevent fractures [99]. The women and GPs were found to view screening positively. Risk assessment using the FRAX tool, compared to usual care, showed no impact on anxiety (State-Trait Anxiety Inventory) and quality of life (EuroQol 5-Dimension tool and the Short-Form Health Survey 12) (*P* > 0.10 for all outcomes). The authors concluded that an effective and cost-effective screening programme to reduce osteoporotic fractures could be implemented in routine care and would be well received by women and GPs [99].

The data to date suggest that assessment of fracture risk by the FRAX tool, with subsequent BMD measurement where indicated, is acceptable to the populations in which it has been used.

#### There should be an agreed policy on the further diagnostic investigation of individuals with a positive test result and on the choices available to those individuals.

The use of risk calculators to determine who should receive an intervention is somewhat different from many established screening programmes. For example, breast tissue calcification might indicate malignancy, but further investigation is required to confirm or refute the diagnosis of breast cancer. In contrast, the patient identified at high risk by a risk calculator arrives there because of the known factors entered into the calculation. In many osteoporosis guidelines, this risk assessment may indicate the need for BMD measurement, if not already undertaken, to provide additional risk information.

In clinical settings, this assessment of BMD, usually by DXA measurements at the lumbar spine and hip, is used not just for risk prediction, but also for diagnosis, selection of patients for treatment and monitoring of patients on treatment. A requirement has been estimated of approximately 11 DXA units per million of the general population to permit implementation of practice guidelines [72], a level which is only achieved in about 60% of countries in the EU [11, 100]. Strategies that target the use of DXA to those with fracture risks at or near an intervention threshold reduce the need for DXA while maintaining the identification of those at high risk. For example, a comparison of the NOGG strategy, comprising risk assessment followed by targeted DXA, with the previous guidance issued by the Royal College of Physicians (RCP) where the presence of a risk factor simply mandated the use of DXA, NOGG identified a similar proportion of women at high risk (average 34.6% vs. 35.7% across all ages), but with lower numbers of scans required at each age [68]. Thus, the NOGG approach required only 3.5 scans at the age of 50 years to identify one case of hip fracture, whereas the previous RCP approach required 13.9. At 75 years, the corresponding numbers were 0.9 and 1.5. Compared to the older strategy, the FRAX-based NOGG strategy used BMD resources more efficiently with lower acquisition costs and lower costs per hip fracture averted [68].

Most clinical guidelines agreement the need for additional radiological, haematological, biochemical and immunological assessments in patients at high risk of fracture or with recently diagnosed osteoporosis [50, 51, 53]. Broadly, these seek to identify or exclude any undetected underlying disease that might contribute further to fracture risk and its management (e.g. identify a reversible or treatable cause such as primary hyperparathyroidism or coeliac disease), and to identify any contraindications to a particular treatment (e.g. renal impairment in the case of bisphosphonates or hypocalcaemia in the use of potent bisphosphonates or denosumab).

In summary, there is much agreement across clinical guidelines on the further diagnostic investigation of individuals at increased risk of fracture and on the treatment choices available to such individuals (see next section).

### The treatment

#### There should be an effective treatment or intervention for patients identified through early detection, with evidence of early treatment leading to better outcomes than late treatment

Approaches to reduce fracture risk incorporate non-pharmacological measures [101, 102] and pharmacological treatments. Of the many factors that influence the risk of fracture, age-related reductions in bone mass and increased likelihood of falling are important contributors [103]. While assessment of falls risk and appropriate interventions aimed at reducing falls risk have been shown to be effective [104], at least in the short term, their impact on the risk of fracture, particularly at the hip, is less certain [105, 106]. For example, in a recent review, multifactorial interventions or single exercise-based interventions were found to reduce falls risk, but the impact on fractures was not significant [107].

In contrast, many randomised, placebo-controlled trials have shown that treatments directed at preventing bone loss and/or improving bone mass can significantly reduce the incidence of fracture [108] at vertebral (relative risk, RR 0.4–0.6) and non-vertebral sites (RR 0.6–0.8), including reducing the incidence of hip fracture by 30–45% [109–111]. Classes of drugs demonstrating efficacy in the prevention of fragility fracture include bisphosphonates (e.g. alendronate, ibandronate, risedronate and zoledronate), selective oestrogen receptor modulators [SERM] (e.g. raloxifene, bazedoxifene), parathyroid hormone peptides and derivatives (e.g. teriparatide and abaloparatide), menopausal hormone therapy (MHT) and humanized monoclonal antibodies (e.g. denosumab and romosozumab). Recent comparative clinical trials have provided evidence of enhanced anti-fracture efficacy of anabolic compared with antiresorptive therapies [112–114], prompting considerations of starting treatment with an anabolic agent in patients at very high risk of fracture as a more appropriate means of rapidly reducing fracture risk [115–117].

The availability of effective therapies has long given rise to considerations of approaches for fracture prevention; for example, almost 20 years ago, a simulation model investigated the cost-effectiveness of a 5-year treatment to prevent hip fracture in women at average risk [118]. Major determinants of cost-effectiveness included the age of the individual and the cost of treatment. Assuming an efficacy of a 35% reduction in relative risk, it was cost-effective to treat all women above the age of 81 years with an intervention cost of $650/year. For cheaper treatments, e.g. $250, it was cost-effective to give treatments at the age of 70 years, and for the cheapest treatment to treat nearly all postmenopausal women. The authors suggested that controlled prospective studies in the apparently healthy population were worthy of consideration, particularly in the elderly. Since then, several randomised, controlled studies have examined the use of osteoporosis interventions in older populations, unselected for osteoporosis. In the Women’s Health Initiative study, the use of MHT was examined in two studies, one in women with an intact uterus and one in women with a history of hysterectomy [119, 120]. The effects on fracture risk were similar between the two studies. In women age 50–79 years with an intact uterus, the use of conjugated equine oestrogen and medroxyprogesterone acetate over a mean of 5.2 years follow-up reduced the incidence of hip fracture by 34% (hazard ratio, HR: 0.66; 95% CI: 0.45–0.98], with a 23% reduction in other osteoporotic fractures (HR: 0.77; 95% CI: 0.69–0.86) [119]. Importantly, this effect was independent of baseline BMD [121] suggesting that treatment efficacy was not dependent on the presence of osteoporosis. A similar BMD-independent effect on fracture risk reduction was observed in a 3-year randomised, placebo-controlled study of the oral bisphosphonate, clodronate, in women age 75 years and older, again unselected for osteoporosis [122]. This study was underpowered for the outcome of hip fractures, but treatment was associated with a 23% reduction in osteoporotic fractures. A post hoc analysis demonstrated that, while the effect was independent of BMD, treatment was more effective in women at higher baseline fracture risk assessed by the FRAX tool [123]. Thus, at a probability of 15% (25th percentile), the relative risk for fracture was reduced by 8% (RR 0.92, 0.69–1.24), whereas at probabilities of 24% and 30% (the 75th and 90th percentiles), the reductions were 27% (RR 0.73, 0.58–0.92) and 38% (HR 0.62, 0.46–0.84) respectively. Several other studies of a variety of osteoporosis therapies have also shown no interaction between treatment efficacy and underlying BMD [124] supporting the use of treatment in those with high fracture risk even before a BMD in the osteoporosis range has been reached.

The success of such population-based approaches does not necessarily imply that risk assessments to target therapies are not worthwhile, since it is likely to be even more cost-effective to target individuals at higher-than-average risk. Certainly, the more expensive the treatment and the cheaper the risk assessment, the stronger the case for selecting high-risk segments of the population. The age at which intervention might be offered depends critically upon the costs of treatment, efficacy and the average risk of hip fracture, as well as additional skeletal and extraskeletal effects. The aforementioned cost-effectiveness analysis in 2001 suggested that strategies aimed at intervention over the age of 70 years required testing [118].

In summary, there is an extensive clinical and economic evidence base for effective bone-targeted treatments that can reduce fracture risk. Unselected population-based studies suggest that fracture risk can be also significantly reduced with such treatments; targeted intervention of relatively inexpensive, safe treatments to those within these populations that have identifiable increased risk will intuitively lead to better outcomes than treatment delayed until after the subsequent occurrence of fracture.

#### There should be agreed evidence-based policies covering which individuals should be offered treatment and the appropriate treatment to be offered

Most clinical guidelines for the management of osteoporosis adopt a case-finding strategy, combining clinical risk factors with measurement of BMD, preferably at the hip, to assess fracture risk and subsequent use of defined intervention thresholds [67]. The majority recommend treatment for osteoporosis in individuals with prior fragility fractures, especially fractures of the spine or hip, without specific need for BMD assessment. For those without prior fractures, intervention thresholds have historically focused on the BMD T score ≤  − 2.5, with treatments reimbursed in many healthcare settings when a BMD-based diagnosis of osteoporosis is confirmed. As noted previously, the realization that BMD-defined osteoporosis has high specificity, but low sensitivity for future fractures has underpinned the movement towards the use of absolute fracture risk to define intervention thresholds, usually calculated by the FRAX tool. There are differences in approach with some guidelines recommending fixed intervention thresholds, while others have proposed age-dependent thresholds or, in a minority of cases, a hybrid of age-dependent and fixed thresholds. In the UK, NOGG developed a guideline on the basis of clinical appropriateness, setting the threshold at the age-specific probability of fracture equivalent to a woman having already sustained a fracture. The latter process is supported by, but not dependent on, the demonstration of cost effectiveness; it avoids inappropriate over-treatment of older individuals and under-treatment of younger individuals.

Cost-effectiveness is accepted in many countries as a primary driver of decisions about who should be offered treatment with particular medications. Such approaches arose from the perceived need to limit access to expensive treatments across a range of diseases. Within osteoporosis, for example, health technology assessments of available treatments in the UK by the NICE have traditionally stratified the risk levels at which treatments could be initiated [125–129]. This stratification was usually based on age, BMD T score and the presence of one or more individual clinical risk factors; the cost-effectiveness thresholds were subsequently assumed by the clinical community to be intervention thresholds. More recently, NICE appraisals have embraced the use of absolute fracture risk, but the continued application of cost-effectiveness to developing thresholds for now inexpensive drugs led to counterintuitive and potentially harmful guidance [130].

In summary, many countries have access to evidence-based policies and guidelines covering which individuals should be offered treatment and the appropriate treatment to be offered.

#### Clinical management of the condition and patient outcomes should be optimised in all healthcare providers prior to participation in a screening programme

**Fig. 2:**
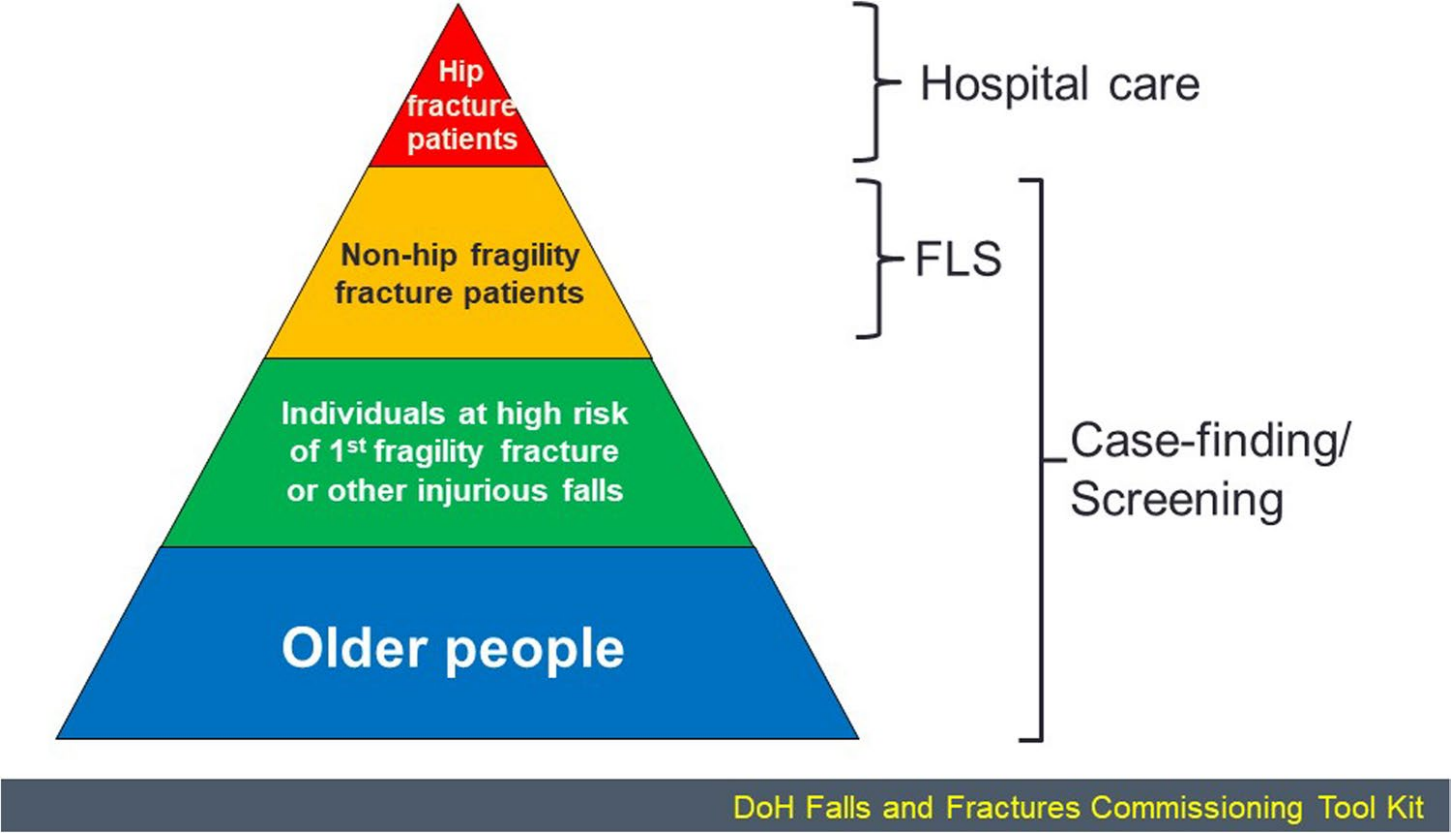
A systematic approach to fracture prevention adapted from that outlined by the Department of Health in the UK. FLS — fracture liaison services

A general approach to reducing the fracture burden in society was outlined by the Department of Health in the UK in 2009 (Fig. 2). This approach recognized the need to start by providing optimal care to those with hip fractures, followed by the provision of services that would identify, investigate and treat those presenting with non-hip fragility fractures (so-called secondary prevention).

A standardized approach to post hip fracture care was first published in a collaboration between the British Orthopaedic Association and British Geriatric Society in 2007 [131], followed soon after by the establishment of the National Hip Fracture Database to audit hospital performance. The database is centrally funded via government but is run independently. Importantly, the programme was subsequently supported by the creation in 2010 of a Best Practice Tariff (BPT) to financially incentivize improved care. Subsequently, NICE incorporated much of this acute hip fracture management initiative into their own guideline in 2011 [132]. The guidelines state that patients should be managed with combined orthopaedic and orthogeriatric care in a hip fracture programme with all patients preferably being seen preoperatively; due to difficulties in accessing orthogeriatric services at weekends, the target is that all patients are reviewed within 72 h from admission. Notably, 30-day mortality fell by 7.6% per year in the 4 years after the introduction of the National Hip Fracture Database compared to a 1.8% per year decrease in the 4 years preceding its introduction [133]. Similar systems are being established in other European countries [134].

In the next tier of the pyramid in Fig. 2, it was recognized that patients presenting with a fragility fracture, related or unrelated to a fall, should be assessed for osteoporosis and receive effective management to improve their bone health and reduce future fracture risk. The preferred solution is the establishment of Fracture Liaison Services, which are usually hospital-based and provide a co-ordinator to identify patients aged 50 and over with a first fracture [135, 136]. The identification is followed by risk assessments and initiation of evidence-based interventions for bone health and falls prevention. Ideally, the service would also monitor adherence and any recurrent events, but this is frequently passed back to primary care services. Treatment includes prescribing bone strengthening medicines, as well as referral to falls risk assessment and prevention services where appropriate. To date, no randomised control trials have demonstrated the effectiveness of FLS in reducing fracture risk, but such trials have shown increased DXA testing, treatment initiation and early adherence in FLS-like settings [137–139]. Observational studies have shown reductions in fracture risk, though somewhat inconsistently, with difficulty in interpreting the results due to a number of biases in such study designs [140]. Many of these biases were overcome in a recent Swedish study, comparing fracture rates before and after FLS implementation, with non-FLS hospitals as an added comparator [141]. The risk of recurrent fracture was 18% lower in the period post FLS implementation compared with the control period (hazard ratio = 0.82, 95% confidence interval [CI] 0.73–0.92, *p* = 0.001), with no change in recurrent fracture rate in the non-FLS hospitals [141]. The number and standard of FLS worldwide continues to increase under the auspices of the International Osteoporosis Foundation’s Capture The Fracture ® programme (https://www.capturethefracture.org/).

There is some overlap between the next two tiers of the strategy shown in Fig. 2, namely individuals at highest risk of fragility fracture and older people, as age itself is a prominent risk factor for fracture. The individuals encompassed by these tiers are largely those where the burden of osteoporosis assessment and management falls on primary care health professionals, and it is this constituency to which the proposed screening approach would be largely directed. There is good evidence that, in this setting, treatment rates are very low. For example, in a recent study, treatment rates in women ≥ 70 years, deemed to be at high risk of fracture, were examined in primary care in 8 European countries [142]. The women were enrolled when visiting their general practitioner, regardless of the reason for attendance, and data captured on fracture risk and osteoporosis treatments. Increased risk of fracture was characterized as one or more of the following, a history of fracture, FRAX 10-year probability of fracture above country-specific thresholds, or a T score ≤  − 2.5 at the spine or hip. In the 3798 enrolled patients, median FRAX probability (calculated without BMD) was 7.2% for hip fracture and 16.6% for major osteoporotic fracture. Overall, 2077 women (55%) met one or more of the criteria for increased risk of fracture (median 10-year probabilities of hip and major osteoporotic fracture 11.2% and 22.8% respectively). An osteoporosis diagnosis was recorded in 804 patients (21%); most (80%) of these were at increased fracture risk. The treatment gap (the proportion at high risk but untreated) was 75%, varying from 53% in Ireland to 91% in Germany [142]. The treatment gap was somewhat lower in women with an osteoporosis diagnosis compared to those without (31% vs. 94% respectively). These data are very similar to osteoporosis treatment gaps assessed in other recent studies [11, 143], and reflect the need for integrated approaches to fracture prevention [144].

As described above, strides are being made in the clinical management of fracture risk, but the impact on patient and healthcare outcomes remains sub-optimal. The proposed screening program, if well implemented, would actually address the current deficiencies in care of a high-risk subgroup within the population, i.e. it should be regarded as a means to optimize healthcare in a greater proportion of those at risk.

### The screening programme

There should be evidence from high quality randomised controlled trials that the screening programme is effective in reducing mortality or morbidity. Where screening is aimed solely at providing information to allow the person being screened to make an “informed choice”, there must be evidence from high quality trials that the test accurately measures risk. The information that is provided about the test and its outcome must be of value and readily understood by the individual being screened.

As stated earlier, the proposed screening programme is based on the randomised, controlled SCOOP study which is described in more detail below [3]. Two additional randomised studies, namely the Risk-stratified Osteoporosis Strategy Evaluation study (ROSE) from Denmark [145] and the SALT Osteoporosis Study (SOS) from the Netherlands [146], have used FRAX-based approaches for population screening and are also discussed below.

#### The SCOOP study

The Screening for Osteoporosis in Older People (SCOOP) study was a multicentre, primary care-based screening programme [3]. Approximately 52,000 women age 70–85 years were identified in 100 primary care practices in England; when those with exclusion criteria which included ongoing treatment for osteoporosis and certain concurrent conditions (e.g. known dementia, terminal illness or recent bereavement), letters of invitation were sent to 38,600 women. Of the latter, 25,571 women did not respond (*n* = 11,068) or declined the invitation (*n* = 13,870), leaving 13,029 eligible participants. A total of 12,483 (95.8% of eligible, 32.3% of those invited) women were finally included in the randomised controlled trial which had a planned follow-up of 5 years.

The steps in the screening protocol are outlined in Table 4. It should be noted that the FRAX risk assessment was on the basis of 10-year hip fracture probability. It was undertaken at study enrolment in the screening arm and, in the control arm, calculated using the baseline questionnaire at the end of the study. In the latter, fracture risk assessment and management were left to normal clinical practice under the care of the GP. In the screening arm, hip fracture probabilities were calculated from the self-completed risk questionnaires; if the 10-year probability was above an age-dependent assessment threshold, the participant was invited for a DXA scan to measure femoral neck BMD. Those with probabilities below the threshold were informed that they were at low risk of hip fracture. In those attending for a DXA scan, the femoral neck BMD was then used in the recalculation of their 10-year hip fracture probability. If the latter value lay at or above an age-dependent intervention threshold, the participant and their GP were informed of a high risk of hip fracture and treatment, largely with oral bisphosphonates, was recommended.

**Table 4:** Comparison of screening strategies across the SCOOP, ROSE and SOS studies in women

Age range	**SCOOP**	**ROSE**	**SOS**
	70–85 years	65–80 years	65–90 years
Number recruited (with baseline FRAX if different)	Control 6250 Screening 6233	Control 17,157 (9326) Screening 17,072 (9279)	Control 5457 Screening 5575
1^st^ screening step			
Assessment	FRAX 10-year hip probability without BMD	FRAX 10-year MOF probability without BMD	FRAX 10-year MOF probability with BMD (plus VFA)
Definition of positive test	Probability ≥ age-dependent assessment threshold	Probability ≥ 15% or more	See treatment criteria below
2^nd^ screening step			
Assessment	DXA measurement of BMD	DXA measurement of BMD	N/A
Treatment criteria	Probability (with BMD) ≥ age-dependent intervention threshold	BMD T-score ≤ − 2.5	Probability ≥ age-dependent thresholds + BMD T score ≤ − 2, or a prevalent vertebral fracture, or met criteria within Dutch guidelines
Performance per prevented fracture			
NNS/NNT (Ost fracture) NNS/NNT (Hip fracture)	133/19 115/17	319/34 281/30	178/32 552/98

**Fig. 3:**
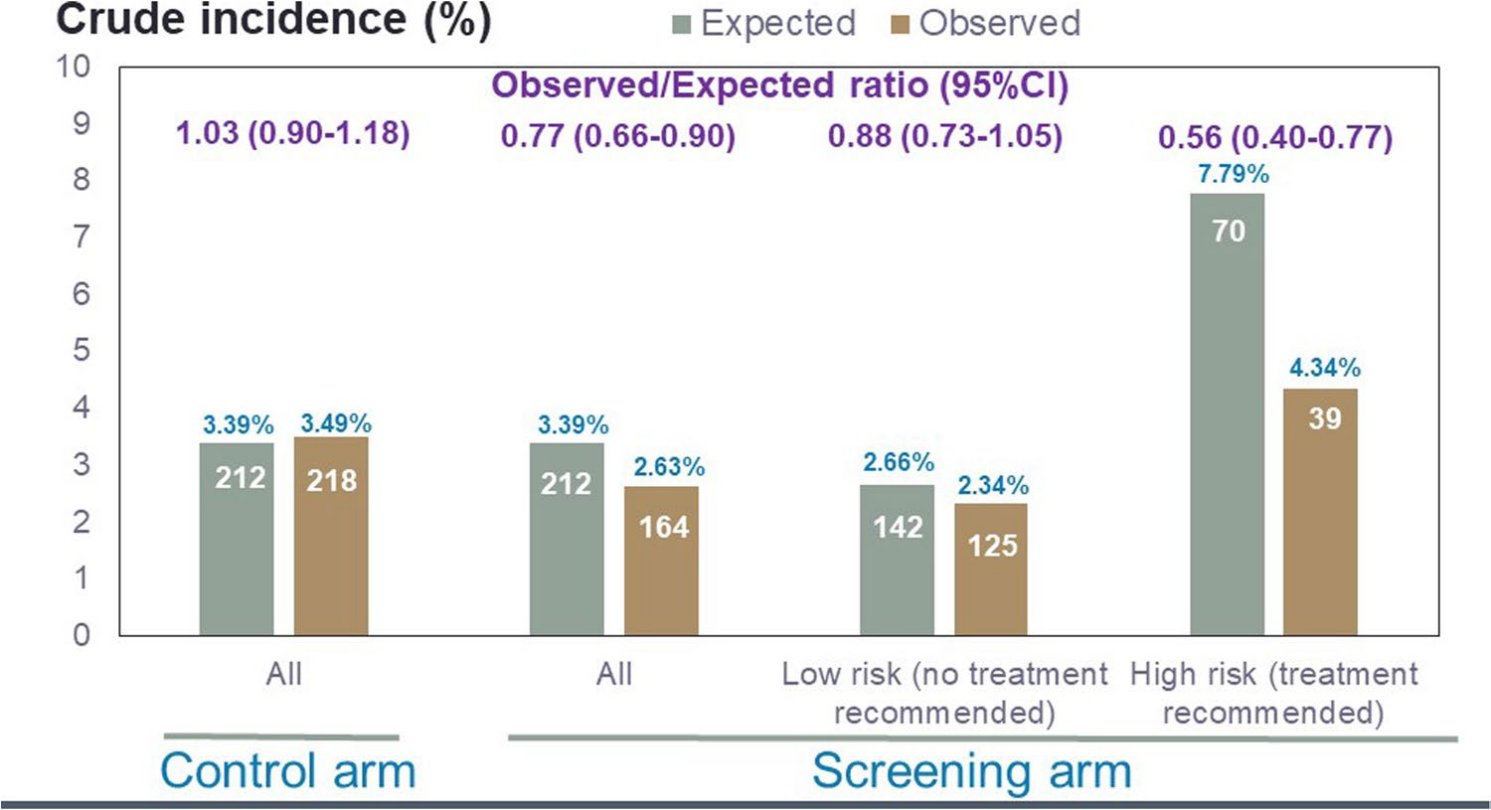
Expected and observed incidences of hip fracture in the control and screening arms of the SCOOP study. The number of hip fractures within each group is shown in white text within the bars. The percentages in blue represent the crude incidences of hip fracture

In the screening arm, only 898 women (14.4% of the group) were identified as at high risk of hip fracture and recommended for treatment. Overall, the screening strategy did not reduce the incidence of all osteoporosis related fracture, (hazard ratio [HR] 0.94, 95% CI 0.85–1.03, *p* = 0.178), but the number of hip fractures in the screening arm was significantly lower than that in the control arm (164 vs. 218 respectively, HR 0.72, 0.59–0.89, *p* = 0.002) [3]. The lack of DXA scanning in the control arm restricted any subsequent comparisons between the two study arms to FRAX probabilities calculated without BMD. A subsequent analysis showed a significant interaction between baseline FRAX hip fracture probability and the effectiveness of screening to reduce the incidence of hip fractures, i.e. the reduction was only seen at high baseline risks associated with the recommendation for treatment [4]. Another approach to determine the contribution of intervention in such a small proportion of the screening arm to the overall hip fracture reduction is shown in Fig. 3. Here, a comparison of observed versus expected number of hip fractures was undertaken; the expected number of hip fractures was calculated from baseline FRAX probabilities using an adaptation of the FRAX tool with a 5-year time horizon to reflect the duration of the SCOOP study.

The adapted FRAX tool predicted that a total of 212 women (3.39%) in the control arm would sustain one or more hip fractures during the SCOOP study; the actual number with incident hip fractures was 218 (3.49%), an observed to expected ratio (O/E ratio) of 1.03 (95%CI 0.90–1.18). In the screening arm, in contrast to an expected number of 212 women with incident hip fractures, only 164 women (2.63%) sustained such fractures, with an O/E ratio of 0.77 (95%CI 0.66–0.90). In the 898 women allocated to the high risk group in the screening arm and recommended for treatment, a total of 70 hip fractures were expected, but only 39 were observed (O/E ratio 0.56, 0.40–0.77). Thus, of the 48 fewer hip fractures than expected, 31 (64.6%) were in this small subgroup (14.4% of the screening group).

The numbers needed to screen (NNS) to prevent one osteoporotic or hip fracture in SCOOP were 133 and 115 respectively; for the same outcomes, the numbers needed to treat (NNT) were 19 and 17 respectively (Table 4).

#### The ROSE study

The ROSE study in southern Denmark was a randomised controlled trial in 34,229 women aged 65–80 years, to investigate the effectiveness of a two-step population-based osteoporosis screening programme using FRAX® to select women for DXA scans [145]. The steps in the screening protocol are outlined in Table 4.

Randomisation to screening (*n* = 17,072) or control (*n* = 17,157) groups took place before the letters of invitation were sent out. Those invited were asked to return a self-completed questionnaire, including the FRAX risk factors. A total of 27,157 women (79.3% of the cohort) returned the questionnaires, but FRAX probabilities were only calculable in 20,905 women (61% of cohort). Following the exclusion of women already on osteoporosis treatment, 18,605 women (54.4%) were included in the next stage of the study where the FRAX 10-year major osteoporotic fracture probability was used to determine the need for a DXA scan to measure BMD (termed the per protocol analysis 1 population). If the FRAX MOF probability was 15% or greater, the women were recommended a DXA scan; this comprised 7056 (76%) out of 9279 women in the screening arm of the study, but DXA scans were only obtained in 5009 women (54% of the high-risk subgroup). Following the DXA scan, osteoporosis treatment was indicated based on criteria defined within Danish guidelines; in brief, treatment was recommended via the GP or specialist clinic if the BMD T score at the spine or total hip was ≤  − 2.5, if a vertebral fracture was detected on lateral spine imaging during the DXA assessment, or if the BMD T score was <  − 1.0 and the woman was receiving ongoing supraphysiologic doses of glucocorticoids [147]. In the screening arm, treatment was recommended in 1236 women and was started in 986 women. Thus, in the screening arm, treatment was recommended in 7.2% of all the women randomised and invited to take part, or 13.3% if the denominator is the number of women completing a FRAX assessment (per protocol analysis 1).

Overall, in 10, there was no difference in incident fractures (MOF, hip fracture, and all fractures) between the screening and control arms during a follow-up of approximately 5 years. In contrast, in the per-protocol analysis 1, while no difference was observed in all incident fractures or MOF, a borderline significant reduction in hip fracture risk was observed in the screened group (169 vs 202 hip fractures, HR 0.82, 95%CI 0.67–1.01, *p* = 0.059). The effect was even more marked in the smaller subgroup who attended for DXA scans (HR 0.74, 95%CI 0.58–0.95, *p* = 0.018), but the latter needs to be interpreted with caution due to the relatively large dropout rate (46%) in those recommended for DXA scans [145].

The NNS to prevent one osteoporotic or hip fracture in ROSE were 319 and 281 respectively; for the same outcomes, the NNT were 34 and 30 respectively (Table 4).

#### The SOS study

SOS in the Netherlands was also a pragmatic randomised controlled trial in women aged 65–90 years with at least 1 clinical risk factor for fracture [146]. The latter included previous fracture after age 50 years, parental hip fracture, BMI < 19 kg/m^2^, rheumatoid arthritis, early menopause (< 45 years of age), malabsorption syndrome, chronic liver disease, type I diabetes mellitus or immobility (severe walking difficulties and/or use of walking aid). Exclusion criteria included a predicted short life expectancy, current or recent osteoporosis treatment, high body weight (> 135 kg) or glucocorticoid use ≥ 7.5 mg prednisone equivalent/day.

From a total pool of almost 54,000 women in primary care, 25,314 completed a baseline questionnaire and 11,032 fulfilling the inclusion/exclusion criteria were entered into the study. Of the 5575 women randomised to the screening arm, the screening procedures were only conducted in 4228 (75.8%). The procedures included DXA, vertebral fracture assessment (VFA), FRAX assessment, falls history and blood tests to exclude secondary osteoporosis. Following this, treatment was recommended in 1417 women (25% of all women randomised to screening or 33.5% of those undergoing the screening procedures). Treatment criteria predominantly comprised a FRAX 10-year MOF probability, calculated with BMD, greater than age-dependent thresholds (these ranged from > 15% in women aged 60–65 years, to > 32% in women aged 85–91 years). This criterion did not mandate treatment if the T score was >  − 2.0; 170 women were excluded from treatment on this basis [148]. Some women also received treatment if they fulfilled the criteria published within Dutch guidelines but did not reach the FRAX-based thresholds; an additional 36 women were treated on this basis [148].

Over a planned study follow up of 3 years, 626 women in the screening group had a fracture compared to 632 in the usual care group (HR 0.97, 95%CI 0.87–1.08). The point estimate of the hazard ratio for other fracture outcomes, including osteoporotic fractures, major osteoporotic fractures and hip fractures was somewhat lower (0.91), but did not reach statistical significance. For example, 133 women sustained hip fractures in the screening arm compared to 143 women in the usual care arm (HR 0.91, 95% CI 0.71–1.15). In an exploratory analysis among participants with a recent fracture (< 2 years before baseline), fewer women sustained hip fractures in the screening arm but the number of events was small (10 versus 25, HR 0.38; 95% CI 0.18–0.79). The authors noted that the outcome of the trial may have been impacted by nonparticipation and medication nonadherence in the screening group.

The NNS to prevent one osteoporotic or hip fracture in SOS were 178 and 552 respectively; for the same outcomes, the NNT were 32 and 98 respectively (Table 4).

### Meta-analysis of FRAX-based screening RCTs

**Fig. 4:**
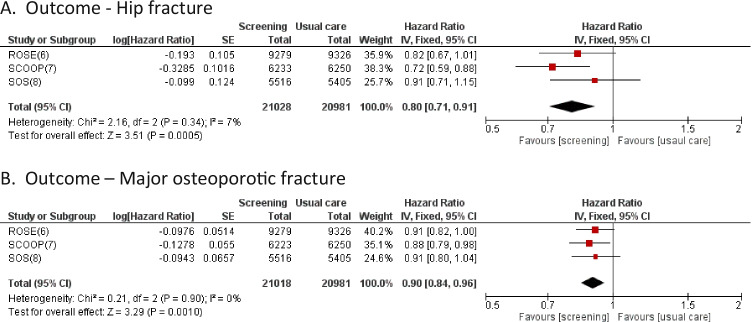
Forest plots of screening for prevention of hip (**A**, adapted from [149]) and major osteoporotic (**B**) fractures versus usual care. Note: From the ROSE study, the data from the first per protocol analysis were used, as these were most comparable to the data from the SCOOP and SOS studies. In **B**, the meta-analysis from [149] has been updated to include the major osteoporotic fracture outcome from the SCOOP study. **A** Outcome — hip fracture, **B** Outcome — major osteoporotic fracture

As part of a systematic review of randomised studies of screening for high fracture risk, the investigators of the SOS study subsequently undertook a meta-analysis of the three studies described in detail above [149]. This analysis comprised a total of 42,009 participants, reflecting all the women participating in the SCOOP and SOS studies, plus all the women providing a FRAX-based assessment at entry to the ROSE study. The latter group was deemed to be comparable to the participants in the other two studies. The proportions receiving treatment recommendations in the studies were 13% in ROSE, 14% in SCOOP and 25% in SOS, with subsequently 11%, 15%, and 18% respectively commencing therapy. Despite these relatively low treatment rates, the meta-analysis showed a statistically significant decrease of 5% in the incidence of osteoporotic fractures (HR 0.95, 95% confidence interval (CI) 0.89–1.00) when comparing screening to usual care. Most notably, there was a 20% decrease in hip fractures (HR 0.80; 95%CI 0.71–0.91) (Fig. 4A). There was also a 9% decrease in major osteoporotic fractures (HR = 0.91; 0.84–0.98), but this analysis didn’t include the outcome of major osteoporotic fractures in 9 (HR 0.88, 0.79–0.98, *p* = 0.018) [150]. Addition of the SCOOP result to the meta-analysis resulted in a similar overall reduction in major osteoporotic fractures (Fig. 4B). There was no difference in all-cause mortality (HR 1.04; 0.95–1.14). It is worth noting that both ROSE and SOS used the FRAX MOF output to target assessment; if the FRAX Hip output had been used as in 9, it is possible that the reduction in hip fractures would have been somewhat greater.

In the pooled cohort, the NNS and NNT for hip fractures were 272 and 28 respectively. The meta-analysis clearly showed population screening to be effective, with the biggest reduction observed in the outcome of hip fracture, leading the authors to conclude that implementation of screening in older women should be considered a serious option.

In summary, three randomised controlled studies and a subsequent meta-analysis have established efficacy for the use of FRAX screening to reduce the risk of hip fracture, as well as other fracture outcomes.

#### There should be evidence that the complete screening programme (test, diagnostic procedures, treatment/ intervention) is clinically, socially and ethically acceptable to health professionals and the public.

None of the steps or assessments in the proposed screening programme is novel. The FRAX questionnaire has been used in routine clinical practice since its launch in 2008. The website alone, which is not the only means of accessing the FRAX calculation, has had approximately 34 million calculations over the last 10 years (site accessed on 16th July 2021). In a recent review, there was widespread usage of the online tool globally [98], with its daily place in the management of patients at risk of fracture reflected in the marked downturn in usage associated with the onset of the global pandemic [151].

Bone mineral density measurement by DXA has been part of routine clinical care of osteoporosis since the 1980s and is a widely accepted technique for both patients and doctors. The radiation dose is low (equivalent to 2–3 days of background radiation) [152], the scanning time is rapid (a few minutes) and the scan does not require the patient to be enclosed or in a tunnel.

A randomized controlled trial of screening using BMD found no evidence of an adverse effect on either quality of life or anxiety [153], while qualitative studies have reported some anxiety (i.e. a low BMD conveying a feeling of weakness) but others have reported more positive views of BMD measurement [154, 155]. A specific assessment of the acceptability of the proposed screening approach used in the SCOOP study has been published [99]. The results showed that women and GPs viewed screening positively, recognizing its potential to improve fracture prevention and future health.

In summary, none of the components of the screening programme are new or untested, with many years of experience suggesting that the programme is clinically, socially and ethically acceptable to health professionals and the public.

#### The benefit gained by individuals from the screening programme should outweigh any harms, for example from overdiagnosis, overtreatment, false positives, false reassurance, uncertain findings and complications.

As noted, qualitative data indicate that the proposed screening programme is not associated with any increase in anxiety. The numbers needed to treat to prevent one hip fracture, derived from the meta-analysis, compare very favourably with those for the use of statins to prevent non-fatal myocardial infarctions or strokes in patients with (https://www.thennt.com/nnt/statins-for-heart-disease-prevention-with-known-heart-disease/) or without (https://www.thennt.com/nnt/statins-for-heart-disease-prevention-without-prior-heart-disease-2/) known heart disease. The screening test identifies a risk or, more accurately in the case of FRAX, a probability, rather than a specific disease or a pre-disease state; the use of false positives and false negatives in this setting is complex. The positive and negative predictive values are probably of most importance to clinicians and patients alike, combined with information on the safety and efficacy of the treatments. There is therefore a need to ensure that the individual patient’s understanding of risk is addressed, that their understanding of the importance of the adverse outcome (i.e. fracture, particularly hip fracture) is good, and the benefits and risks of the treatment or intervention are clear. All the treatments used in 8 are licensed and many are long-established with very well characterised data on efficacy and side effects. Public and individual awareness of rare side effects of osteoporosis treatments, namely atypical femoral fractures (AFF) and osteonecrosis of the jaw (ONJ), can impact on treatment uptake despite occurring at very low rates in the treated osteoporosis population (approximately 1 in 1000–100,000 for AFFs and 1 in 10,000–100,000 for ONJ) and usually only after several years of exposure [156–159]. The risks of these outcomes are far outweighed by the number of fractures at the hip and other sites prevented by treatment [156, 160].

#### The opportunity cost of the screening programme (including testing, diagnosis and treatment, administration, training and quality assurance) should be economically balanced in relation to expenditure on medical care as a whole (value for money). Assessment against these criteria should have regard to evidence from cost benefit and/or cost effectiveness analyses and have regard to the effective use of available resource.

Several cost-effectiveness analyses of population screening strategies in osteoporosis have been published over the last 20 years, but relatively few have been tested in randomized controlled trials [153, 161]. Of the three recent randomised controlled studies using FRAX, only the SCOOP study has published cost-effectiveness analyses to date. The first of these used a within-trial design limited to the time frame of the trial (5 years), with questionnaires (EQ-5D-3L) administered to document self-reported quality of life (QoL) at 6-month intervals during the first year and then at annual intervals used to derive QALYs[6]. The screening arm had a non-significant average incremental QALY gain of 0.0237 (95% CI –0.0034 to 0.0508) for the 5-year follow-up, with an incremental cost per QALY gained of £2772 compared with the control arm. Cost-effectiveness acceptability curves indicated a 93% probability of the intervention being cost-effective at values of a QALY greater than £20,000. The authors concluded that the analysis demonstrated that a systematic, community-based screening programme of fracture risk in older women in the UK represents a highly cost-effective intervention. The most recent analysis used a more established health economic Markov model study design, whereby disutilities of fractures, derived from other quality of life studies, were applied to the women sustaining incident fractures [162]. In addition, the cost-effectiveness analysis modelled a linear offset of effect of the screening programme and calculated the cost-effectiveness over a longer time horizon (remaining lifetime). The model was populated with costs related to drugs, administration and screening intervention derived from the SCOOP study with fracture risk in the control and screening arms corresponding to the observed risk observed in the study. The analysis reported that screening of 1000 patients would save 9 hip fractures and 20 non-hip fractures over the remaining lifetime (mean 14 years) and save costs (£286) in comparison with usual management. The analysis strongly suggests that there are no opportunity costs to a screening programme based on the SCOOP study, and its implementation could actually be cost saving.

Thus, the opportunity costs of the screening programme are low or even cost-saving. They are comparable to or better than many public health measures [163] or other established screening programmes [164, 165]

#### All other options for managing the condition should have been considered (such as improving treatment or providing other services), to ensure that no more cost effective intervention could be introduced or current interventions increased within the resources available.

Current approaches to fracture prevention are addressed in the section on clinical management of the condition above. In the Department of Health guidance in the UK, a focus is given to interventions that will reduce falls risk and/or fracture risk [166]. Falls reduction strategies are also recommended in many international guidelines as a means of reducing falls-related injuries, of which fracture is one of the most serious consequences, especially hip fracture. While assessment of falls risk and appropriate interventions aimed at reducing falls risk has been shown to be effective, at least in the short term, their impact on the risk of hip fracture is less certain [104, 107]. Furthermore, the reduction in hip fractures in the SCOOP study was not mediated by any impact of the screening programme on falls risk [167].

In summary, while falls prevention measures should continue to be included in individual patient management, the role for bone-targeted treatments should be prioritised.

## Conclusion

In this paper, we have sought to assess the potential for a screening programme to identify women at increased risk of hip fracture. We have approached this by proposing a programme based on that used successfully in the SCOOP study and then addressing 15 of the 19 criteria established by the UK National Steering Committee for the assessment of such a programme (Table 5). Notably, evidence addressing these criteria were reviewed for osteoporosis screening by the UK NSC in 2019 (evidence cut-off September 2018), following an external review, and concluded screening could not currently be recommended [168, 169]. A similar conclusion was reached in an assessment under an EU Health Technology Assessment report also published in 2019 [170]. These conclusions were largely based on areas of continuing uncertainty which may be seen as relevant to screening in the UK and beyond. For example, uncertainty remained about the accuracy of screening tests in women who would be included in a population screening programme. We would argue that there is compelling evidence from systematic reviews and individual studies that the accuracy for future hip fractures, as reflected by the AUCs, is appropriate for a clinical tool for the prediction of future events as opposed to the detection of existing or early disease as in other screening programmes [171].

Uncertainty has also been expressed about the effect of treatment and changes in lifestyle on some types of fracture, and on fracture risk in women identified as being at risk of fracture through screening. It is widely recognised that the effect of anti-osteoporosis treatments differs across skeletal sites. For example, analyses consistently show the largest reductions in vertebral fractures (50–70%), intermediate reductions in hip fractures (40–50%) and somewhat lower reductions in non-vertebral, non-hip fractures (20–25%). Both the UK NSC and the EU HTA reports acknowledged that there was evidence of potential benefit for hip fractures [168–170], though neither conducted a meta-analysis that combined the similar populations in both the SCOOP and ROSE studies (i.e. confined to those who returned a baseline risk questionnaire). It is also important to bear in mind that unlike randomised controlled trials of treatments versus placebo, the recent studies of screening have examined strategies rather than treatment; importantly, the proportion targeted for treatment in these studies was small (e.g. 14% in SCOOP) meaning that the majority of participants in both arms of the studies did not receive any therapeutic intervention. A 25% reduction in non-vertebral, non-hip fractures during treatment would be manifest by only a 3.5% reduction in the whole cohort where treatment was just targeted to 14% of the population. It should also be noted that the proposed screening program, predicated on FRAX 10-year probability of hip fracture, targets interventions to individuals with a higher risk of a fracture that is more reversible by osteoporosis treatments. A previous post hoc analysis within the SCOOP study showed that the reductions in hip fractures at baseline high FRAX hip probability are of a similar magnitude to that observed in randomised controlled studies of antiresorptive treatments, an observation that is supported by the analysis presented in this report of the 55% reduction in hip fractures in the small high-risk cohort. We would conclude that there is good evidence that women identified by screening are responsive to appropriate management with anti-osteoporosis agents, particularly with regard to prevention of hip fractures as reflected in the recent meta-analysis.

Finally, uncertainties have been raised about how much added benefit would be gained by population screening over usual care, and the cost-effectiveness of a population screening programme. While none of the screening studies were designed to directly capture impacts on morbidity and mortality, hip fractures are well documented to result in significant morbidity, hospitalisation, surgery and prolonged rehabilitation with a significant number of patients not returning to their pre-fracture level of mobility and independence. The proposed strategy combines a low-cost assessment with targeted intervention using low-cost generic treatments, predominantly oral bisphosphonates; the subsequent 28% reduction in the incidence of hip fractures is a highly impactful strategy compared to usual care. Even more importantly, the standard cost-effectiveness analysis demonstrates that the strategy dominates usual care, i.e. it is actually cost saving. Future health economic modelling should enable assessment of different screening strategies for hip fracture risk, including the wider utilisation of BMD as part of the strategy.

The major limitation of this report, perhaps, is that it is not based on a systematic review to address the available literature on the areas addressed by each specific criterion, and the selected randomised controlled trials excluded other non-FRAX approaches. For example, we did not include a study that addressed screening for prevalent vertebral fractures as the latter was of relatively short duration (1 year) and had medication uptake as the main endpoint [172]. However, the expert panel comprises a wide, in-depth knowledge and expertise in the fields of risk assessment, guidelines, treatment and health economics of osteoporosis. The approach used attempted to measure the level of consensus on the various criteria from a wide variety of geographic and healthcare system settings. The report is meant to serve as a basis for engagement and discussion with policy makers and payers around the potential for screening in osteoporosis with a particular focus on high hip fracture risk.

From the evidence reviewed in this report, we would contend that the case for screening to identify women at increased risk of hip fracture is now made and that future research should focus on strategies for optimal implementation of this approach across different healthcare systems and settings. We would certainly conclude that any transition towards screening that leads to improved identification of hip fracture risk in older women in primary care (e.g. enhanced case-finding) will undoubtedly have positive impacts on the clinical, personal and economic burden of this most serious of osteoporotic fractures.

## Appendix

**Table 5:** Current UK criteria used by the UK NSC for assessment of a screening programme**.** For each criterion, a decision has been made as to whether screening for hip fracture risk using the proposed programme would satisfy the criterion needs. The numbers in parentheses represent the number of responses saying yes, in part or no for each criterion (a total of 20 experts responded to the survey). The final four criteria were considered beyond the scope of this paper

	**Criterion**	**Met (✓) or not met (χ)** **(Yes, in part, no)**
The condition	• The condition should be an important health problem	✓ (17,3,0)
	• The epidemiology and natural history of the condition, including development from latent to declared disease, should be adequately understood and there should be a detectable risk factor, disease marker, latent period or early symptomatic stage	✓ (18,2,0)
	• All the cost-effective primary prevention interventions should have been implemented as far as practicable	✓ (12,7,1)
The test	• There should be a simple, safe, precise and validated screening test	✓ (16,3,1)
	• The distribution of test values in the target population should be known and a suitable cut-off level defined and agreed	✓ (17,2,1)
	• The test should be acceptable to the population	✓ (19,1,0)
	• There should be an agreed policy on the further diagnostic investigation of individuals with a positive test result and on the choices available to those individuals	✓ (17,3,0)
The treatment	• There should be an effective treatment or intervention for patients identified through early detection, with evidence of early treatment leading to better outcomes than late treatment	✓ (16,3,1)
	• There should be agreed evidence-based policies covering which individuals should be offered treatment and the appropriate treatment to be offered	✓ (19,1,0)
	• Clinical management of the condition and patient outcomes should be optimised in all healthcare providers prior to participation in a screening programme	✓ (18,1,1)
The screening programme	• There should be evidence from high-quality randomised controlled trials that the screening programme is effective in reducing mortality or morbidity. Where screening is aimed solely at providing information to allow the person being screened to make an informed choice (e.g. Down’s syndrome, cystic fibrosis carrier screening), there must be evidence from high-quality trials that the test accurately measures risk. The information that is provided about the test and its outcome must be of value and readily understood by the individual being screened	✓ (16,3,1)
	• There should be evidence that the complete screening programme (test, diagnostic procedures, treatment/intervention) is clinically, socially and ethically acceptable to health professionals and the public	✓ (19,1,0)
	• The benefit from the screening programme should outweigh the physical and psychological harm (caused by the test, diagnostic procedures and treatment)	✓ (19,1,0)
	• The opportunity cost of the screening programme (including testing, diagnosis and treatment, administration, training and quality assurance) should be economically balanced in relation to expenditure on medical care as a whole (i.e. value for money). Assessment against these criteria should have regard to evidence from cost benefit and/or cost-effectiveness analyses and have regard to the effective use of available resource	✓ (16,4,0)
	• All other options for managing the condition should have been considered (e.g. improving treatment, providing other services), to ensure that no more cost-effective intervention could be introduced or current interventions increased within the resources available	✓ (14,4,2)
	• There should be a plan for managing and monitoring the screening programme and an agreed set of quality assurance standards	Beyond scope
	• Adequate staffing and facilities for testing, diagnosis, treatment and programme management should be available prior to the commencement of the screening programme	Beyond scope
	• Evidence-based information, explaining the consequences of testing, investigation and treatment, should be made available to potential participants to assist them in making an informed choice	Beyond scope
	• Public pressure for widening the eligibility criteria for reducing the screening interval, and for increasing the sensitivity of the testing process, should be anticipated. Decisions about these parameters should be scientifically justifiable to the public	Beyond scope
